# Uniform efficacy of SGLT2 inhibitors across the ejection fraction spectrum and in high-risk patients with HFpEF: a prespecified pooled analysis

**DOI:** 10.3389/fcvm.2026.1790378

**Published:** 2026-03-12

**Authors:** Xiaomin Xue, Ziqiang Yu, Muhua Dai, Tianjie Zhang

**Affiliations:** 1 Institute of Basic Experiments, Zhejiang Academy of Traditional Chinese Medicine, Hangzhou, China; 2 Department of Neurology, Tongde Hospital of Zhejiang Province, Hangzhou, China; 3 Department of Critical Care Units, Tongde Hospital of Zhejiang Province, Hangzhou, China; 4 Department of Cardiology, Tongde Hospital of Zhejiang Province, Hangzhou, China

**Keywords:** heart failure with preserved ejection fraction (HFpEF), Left ventricular ejection fraction (LVEF), Patient subgroups, Sodium-glucose cotransporter 2 (SGLT2) inhibitors, treatment heterogeneity

## Abstract

**Background:**

Heart failure with preserved ejection fraction (HFpEF) accounts for over half of all heart failure cases and imposes a high symptom burden. Although SGLT2 inhibitors are guideline-recommended, it remains uncertain whether their efficacy is uniform across the entire LVEF spectrum and in high-risk populations like recently hospitalized patients.

**Objective:**

To definitively assess the consistency of SGLT2 inhibitor efficacy and safety across LVEF subgroups and in extended, high-risk HFpEF populations through a prespecified pooled analysis.

**Methods:**

This trial-level pooled analysis included 12,251 HFpEF patients (LVEF >40%) from the EMPEROR-Preserved and DELIVER trials. Prespecified subgroups were defined by baseline LVEF (<50%, 50-59%, ≥60%), hospitalization status, and HFimpEF. The primary endpoint was cardiovascular death or heart failure hospitalization. Treatment effect consistency was assessed using an inverse-variance weighted fixed-effects model, with heterogeneity quantified by I^2^ and interaction tested.

**Results:**

SGLT2 inhibitors significantly reduced the risk of the primary endpoint by 20% vs. placebo (HR 0.80, 95% CI 0.73–0.87, *P* < 0.001). This benefit was consistent across all LVEF subgroups (<50%: HR 0.76; 50-59%: HR 0.79; ≥60%: HR 0.82; interaction *P* = 0.690) and extended to key high-risk subgroups: recently hospitalized patients in DELIVER (HR 0.74) and those with HFimpEF (HR 0.71). No new safety signals were identified.

**Conclusion:**

This analysis confirms a uniform class effect of SGLT2 inhibitors in HFpEF, with consistent cardiovascular protection regardless of LVEF or high-risk clinical status. These findings solidify their role as foundational therapy and support a universal treatment strategy across the ejection fraction spectrum.

**Systematic Review Registration:**

https://www.crd.york.ac.uk/prospero/display_record.php?ID=CRD420261277529, PROSPERO CRD420261277529.

## 1 Introduction

Heart failure with preserved ejection fraction (HFpEF) accounts for over half of all heart failure cases in the United States, and its prevalence is rising (1). Individuals diagnosed with HFpEF frequently experience a substantial symptom burden, pronounced functional limitations, and a marked deterioration in quality of life (2, 3). Consistent evidence from the landmark EMPEROR-Preserved and DELIVER trials demonstrates that Sodium-glucose cotransporter 2 (SGLT2) inhibitors (empagliflozin/dapagliflozin) significantly reduce the risk of the primary composite endpoint in HFpEF patients, leading to their recommendation in international guidelines (4–6). Nevertheless, a critical unresolved question persists: whether this therapeutic efficacy remains consistent across the entire spectrum of left ventricular ejection fraction (LVEF) and extends to broader, more heterogeneous patient populations.

Although the overall results of the two trials were highly concordant, the evidence for efficacy in the subgroup with higher LVEF (≥60%) differed: DELIVER showed a clear benefit (hazard ratio [HR] 0.79, 95% confidence interval [CI] 0.64–0.97) (4), whereas the result from EMPEROR-Preserved did not reach statistical significance (HR 0.87, 95% CI 0.69–1.10) (5). This disparity has raised concerns about a potential attenuation of benefit with increasing ejection fraction. Additionally, while the DELIVER trial enrolled broader populations including hospitalized/recently hospitalized patients and those with heart failure with improved ejection fraction (HFimpEF) (4), the efficacy in these clinically important subgroups remains to be clearly established. Subgroup analyses within individual trials are underpowered to provide definitive conclusions regarding the consistency of treatment effects across these subgroups.

To address these uncertainties, we conducted a prespecified, study-level pooled analysis of the EMPEROR-Preserved and DELIVER trials. The primary objectives were: (1) to precisely quantify the treatment effect of SGLT2 inhibitors across key LVEF subgroups (<50%, 50–59%, and ≥60%); (2) to formally test for interaction to assess whether treatment effects differ significantly between these subgroups; and (3) to evaluate efficacy in hospitalized/recently hospitalized patients and in those with HFimpEF. This prespecified pooled analysis was therefore designed to provide definitive evidence regarding the uniformity of the SGLT2 inhibitor class effect across the broad HFpEF population.

## 2 Methods

This study was a prespecified, trial-level pooled analysis of two pivotal phase III trials (EMPEROR-Preserved and DELIVER). The protocol was prospectively registered with PROSPERO (Registration number: CRD420261277529). The aim was to systematically evaluate the heterogeneity of treatment effects of SGLT2 inhibitors across key subgroups in HFpEF, thereby providing precise, high-level evidence for clinical practice.

### 2.1 Data source and trial design

This prespecified, trial-level pooled analysis used data from the EMPEROR-Preserved and DELIVER trials. Both were registered, phase III, randomized, placebo-controlled trials evaluating SGLT2 inhibitors in patients with heart failure and a LVEF >40%. Their primary results have been published (4, 5).

The EMPEROR-Preserved trial (empagliflozin; NCT03057951) enrolled 5,988 symptomatic patients with LVEF >40%. The DELIVER trial (dapagliflozin; NCT03619213) enrolled 6,263 symptomatic patients with LVEF >40% and evidence of structural heart disease or elevated natriuretic peptides; it also prospectively included a subgroup with HFimpEF. In each trial, patients were randomized to receive either the respective SGLT2 inhibitor (10 mg/day) or placebo. Median follow-up was 26.2 months for EMPEROR-Preserved and 27.6 months (approximately 2.3 years) for DELIVER.

For this prespecified pooled analysis, study-level data for key subgroups and endpoints were extracted directly from the primary publications and Supplementary Appendices of the EMPEROR-Preserved and DELIVER trials (4, 5). This included baseline characteristics, event counts, and patient numbers for prespecified subgroups such as LVEF categories (<50%, 50-59%, ≥60%) and special populations (e.g., recently hospitalized patients). The extracted data were then harmonized and pooled by the authors of the present analysis, resulting in a combined analytic sample of 12,251 patients with LVEF >40%. This approach ensured consistent definitions for the analytical population and the primary composite endpoint across both trials.

### 2.2 Study population and subgroup definitions

The population for this pooled analysis consisted of all randomized HFpEF patients included in the primary analysis sets of both trials. To assess heterogeneity of treatment effect, we prespecified the following key subgroups based on baseline LVEF for analysis: guided by the refined classification of heart failure based on LVEF in current guidelines (7), this study defined LVEF <50%, 50–59%, and ≥60% as key subgroups. Furthermore, to investigate the treatment effect in a “broader population”, we specifically prespecified analyses for the following two groups: hospitalized or recently hospitalized patients (patients hospitalized for heart failure at randomization or within 30 days prior to randomization); and patients with HFimpEF, defined as baseline LVEF >40% but a previous LVEF ≤40%. This HFimpEF subgroup was included only in the DELIVER trial. Data for these subgroups were sourced directly from the respective trial's primary or supplementary publications.

### 2.3 Study endpoints

The primary endpoint for this study was consistent with that of the two original trials, namely the composite of cardiovascular death or hospitalization for heart failure. Secondary endpoints included the individual components (cardiovascular death, hospitalization for heart failure) and all-cause mortality. All endpoints adhered to the uniformly defined event adjudication standards of the two trials.

### 2.4 Statistical analysis

This analysis aimed to pool the effect sizes from the two trials within the prespecified subgroups. Data on primary endpoint events and patient counts for each prespecified subgroup were obtained from the primary publications and Supplementary Materials of each trial. First, the natural logarithm of the hazard ratio (logHR) and its standard error (SE) were calculated within each study. To directly address the primary aim of testing for consistency of effects across subgroups (i.e., to test for heterogeneity) rather than estimating a global effect, pooled hazard ratios and 95% CIs were estimated. Given this objective and the minimal anticipated heterogeneity between the two large, harmonized trials, we employed an inverse-variance weighted fixed-effects model. Overall heterogeneity across estimates was assessed using Cochran's *Q* test, with results expressed as I^2^.

To formally test for differences in treatment effect across the prespecified LVEF subgroups (<50%, 50–59%, and ≥60%), a meta-regression analysis was performed. The P-value for interaction (*P* = 0.690) reported in this study was derived from this meta-regression model, comparing the pooled treatment effects among these LVEF categories. This value was not directly reported in the source trial publications but was calculated as part of the present pooled analysis. All analyses were conducted using R software (version 4.5.2) with the metafor package. A two-sided P-value < 0.05 was considered statistically significant.

## 3 Results

A total of 12,251 HFpEF patients were included in this pooled analysis. The baseline characteristics were well-balanced between the SGLT2 inhibitor group and the placebo group, both overall and within each trial (Table 1).

**Table 1:** Baseline characteristics of patients in the EMPEROR-preserved and DELIVER trials.

Characteristic	EMPEROR-preserved (*N* = 5988)	DELIVER (*N* = 6263)
Demographics		
Age (years), mean ± SD	71.8 ± 9.6	71.6 ± 9.5
Female, *n* (%)	2,672 (44.6)	2,756 (44.0)
Race, *n* (%)		
White	4,720 (78.8)	4,705 (75.1)
Asian	1,009 (16.8)	1,316 (21.0)
Black	194 (3.2)	163 (2.6)
Other	65 (1.1)	79 (1.3)
Geographic region, *n* (%)		
North America	1,052 (17.6)	940 (15.0)
Latin America	1,442 (24.1)	1,681 (26.8)
Europe	2,531 (42.3)	2,436 (38.9)
Asia/Pacific	963 (16.1)	1,206 (19.3)
Clinical characteristics		
NYHA functional class III/IV, *n* (%)	Approximately 25%^a^	1,564 (25.0)
LVEF, mean	54%	54%
LVEF distribution, *n* (%)		
LVEF <50%	1,983 (33.1)	2,116 (33.8)
LVEF 50%–59%	2,058 (34.4)	2,256 (36.0)
LVEF ≥60%	1,947 (32.5)	1,891 (30.2)
Ischemic etiology, *n* (%)	2,178 (36.4)	2,365 (37.8)
Prior HF hospitalization, *n* (%)^b^	1,369 (22.9)	2,012 (32.1)

^a^ The primary paper of EMPEROR-Preserved did not directly report the exact percentage of patients in NYHA class III/IV; it was commonly described as “approximately 25%”.

^b^ Prior HF hospitalization: In EMPEROR-Preserved, this refers to hospitalization for HF within 12 months prior to randomization; in DELIVER, it includes any prior HF hospitalization (not limited to 30 days). The percentage for DELIVER is derived from the reported history of HF hospitalization (*n* = 2,012).

A total of 12,251 HFpEF patients were included in this pooled analysis. The baseline characteristics were well-balanced between the SGLT2 inhibitor group and the placebo group, both overall and within each trial. Detailed baseline demographic and clinical characteristics are presented in Table 1, and baseline comorbidities, biomarkers, medications, and special populations are presented in Table 2.

**Table 2:** Baseline comorbidities, biomarkers, medications, and special populations.

Characteristic	EMPEROR-preserved (*N* = 5988)	DELIVER (*N* = 6263)
Key comorbidities		
Type 2 diabetes, *n* (%)	2,938 (49.1)	2,806 (44.8)
Hypertension, *n* (%)	5,295 (88.4)	5,571 (88.9)
History of atrial fibrillation, *n* (%)	3,388 (56.6)	3,688 (58.9)
Chronic kidney disease (eGFR <60 mL/min/1.73 m^2^), *n* (%)	2,990 (49.9)	2,805 (44.8)
Biomarkers and laboratory		
Median NT-proBNP, pg/mL	946 (Sinus rhythm)/994 (Atrial fibrillation)	911 (Sinus rhythm)/1,433 (Atrial fibrillation)
Mean eGFR, mL/min/1.73 m^2^	60.6	60.8
Medications at baseline		
ACEi/ARB/ARNI, *n* (%)	4,866 (81.3)	5,106 (81.5)
Beta-blocker, *n* (%)	5,111 (85.4)	5,367 (85.7)
MRA, *n* (%)	2,282 (38.1)	2,635 (42.1)
Diuretic, *n* (%)	5,118 (85.5)	5,445 (86.9)
Special populations		
HF hospitalization within 30 days prior to randomization, *n* (%)	Not specifically reported	654 (10.4)
HF hospitalization within 12 months prior to randomization, *n* (%)^a^	1,369 (22.9)	Not specifically reported
HFimpEF, *n* (%)	Not included	1,151 (18.4)

^a^ Definitions for recent hospitalization differ between trials: DELIVER prospectively defined patients hospitalized for HF at or within 30 days prior to randomization; EMPEROR-Preserved prespecified the subgroup of patients hospitalized for HF within 12 months prior to randomization.

### 3.1 Efficacy on the primary endpoint

In the overall pooled population, SGLT2 inhibitors were associated with a 20% lower risk of the primary composite endpoint (cardiovascular death or hospitalization for heart failure) compared with placebo (HR 0.80, 95% CI 0.73–0.87; *P* < 0.001). The benefit was directionally consistent in both trials.

### 3.2 Efficacy by left ventricular ejection fraction subgroups

**Figure 1:**
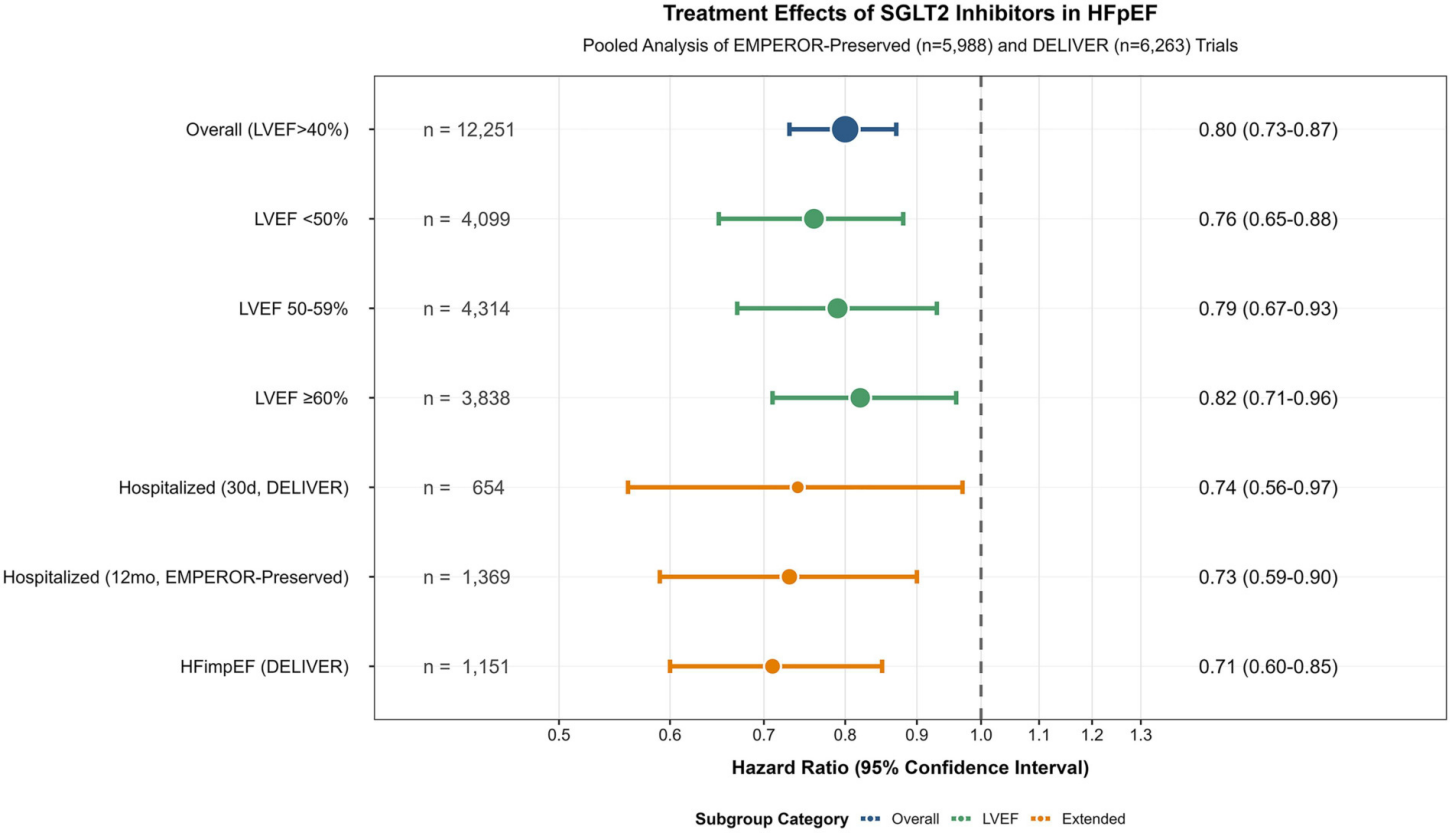
Forest plot of treatment effect across prespecified subgroups. Data are presented as hazard ratio (95% confidence interval). A hazard ratio <1.0 favors SGLT2 inhibitors. Pooled using a fixed-effects model. Heterogeneity across all subgroups: I^2^ = 0%, *P* = 0.990. The *P*-value for interaction across LVEF subgroups (<50%, 50-59%, ≥60%) was 0.690 (from meta-regression). LVEF, left ventricular ejection fraction; HFimpEF, heart failure with improved ejection fraction.

Prespecified subgroup analysis showed consistent treatment effects across LVEF categories (Figure 1; Table 3). SGLT2 inhibitors significantly reduced the risk of the primary composite endpoint in all LVEF subgroups: <50% (HR 0.76, 95% CI 0.65-0.88), 50-59% (HR 0.79, 95% CI 0.67-0.93), and ≥60% (HR 0.82, 95% CI 0.71-0.96). No significant heterogeneity was observed across the LVEF spectrum (P for interaction = 0.690).

**Table 3:** Hazard ratios for the primary composite endpoint by baseline LVEF subgroup.

LVEF subgroup	Number of patients (*n*)	HR	95% CI	Interaction *P* value
Overall (LVEF >40%)	12,251	0.80	0.73–0.87	–
LVEF <50%	4,099	0.76	0.65–0.88	0.690
LVEF 50-59%	4,314	0.79	0.67–0.93	
LVEF ≥60%	3,838	0.82	0.71–0.96	

The *P*-value for interaction is derived from meta-regression comparing the treatment effect in each subgroup against the reference group (LVEF 50%–59%). The overall test for heterogeneity across all three subgroups yielded *P* = 0.690.

### 3.3 Efficacy in extended populations

As shown in Figure 1, treatment effects were also consistent in extended, high-risk populations, including hospitalized/recently hospitalized patients and those with HFimpEF.

In the DELIVER trial, treatment reduced the risk of the primary endpoint by 26% (HR 0.74, 95% CI 0.56–0.97) among patients who were hospitalized for heart failure at the time of randomization or within the prior 30 days (*n* = 654). Although the EMPEROR-Preserved trial did not prespecify an identically defined subgroup, its prespecified subgroup of patients with a heart failure hospitalization within 12 months prior to randomization (*n* = 1,369) showed a consistent and significant 27% risk reduction (HR 0.73, 95% CI 0.59-0.90).

Given the differing definitions of “recent hospitalization” (30 days in DELIVER vs. 12 months in EMPEROR-Preserved), a formal quantitative pooling of these subgroups was not performed. Nevertheless, the qualitatively similar and significant risk reduction observed under both stringent (30-day) and broader (12-month) high-risk definitions reinforces the robustness of the treatment effect in recently hospitalized HFpEF patients.

### 3.4 Secondary endpoints and safety analysis

For the key secondary endpoint, SGLT2 inhibitors showed a clear and consistent benefit in reducing the risk of hospitalization for heart failure (pooled HR 0.75, 95% CI 0.66–0.85). Although a trend toward reduction was observed for cardiovascular death (pooled HR 0.90, 95% CI 0.78–1.04), it did not reach statistical significance. Results for all-cause mortality were similar (pooled HR 0.97, 95% CI 0.87–1.08).

Results for the key secondary endpoint of hospitalization for heart failure, cardiovascular death, and all-cause mortality are presented below. Regarding safety, the pooled analysis identified no new safety signals. No significant differences were observed between the SGLT2 inhibitor group and the placebo group in the incidence of serious adverse events, adverse events leading to drug discontinuation, or events of special interest (such as hypotension, acute kidney injury, or ketoacidosis). This safety profile is consistent with the known class profile of SGLT2 inhibitors and aligns with the overall safety demonstrated in large cardiovascular outcome trials for this drug class (8).

## 4 Discussion

### 4.1 Key findings

This prespecified, trial-level pooled analysis of the EMPEROR-Preserved and DELIVER trials was designed to resolve uncertainties regarding the consistency of SGLT2 inhibitor efficacy across the LVEF spectrum and in broader HFpEF populations. Our findings provide definitive evidence that the benefit of this drug class is remarkably uniform. Most notably, the reduction in cardiovascular risk was consistent across the entire continuum of left ventricular ejection fraction, with no evidence of diminishing efficacy even at higher LVEF (≥60%) (P for interaction = 0.690). This uniformity of effect extends beyond systolic function to encompass clinically relevant, high-risk patient profiles. Specifically, the benefit shows no attenuation in those recently hospitalized for heart failure or in patients with HFimpEF. Furthermore, this broad efficacy is achieved without compromising the established favorable safety profile of SGLT2 inhibitors. We used a fixed-effects meta-analysis model as our primary aim was to test the consistency of treatment effects across subgroups, and statistical heterogeneity was minimal (I^2^ = 0%). Collectively, these results strongly support a class effect and foundational therapeutic role for SGLT2 inhibitors in HFpEF. It is important to note, however, that the observed benefit was driven primarily by a reduction in heart failure hospitalizations, with neutral effects on cardiovascular and all-cause mortality—a distinction that informs the precise clinical positioning of this therapy in HFpEF.

### 4.2 Interpretation and clinical implications

This analysis clarifies a significant benefit of SGLT2 inhibitors in patients with LVEF ≥60%, showing an 18% risk reduction in this subgroup (HR 0.82, 95% CI 0.71–0.96). This precise estimate effectively resolves the uncertainty previously raised by the EMPEROR-Preserved data alone (HR 0.87, 95% CI 0.69–1.10) (5). An interaction P-value of 0.690 strongly supports the hypothesis of a consistent treatment effect across the entire LVEF spectrum, echoing the observed “class effect” of SGLT2 inhibitors in patients with HFrEF (9). Although the point estimates for the primary endpoint showed a numerical attenuation with increasing LVEF (HR 0.76 for LVEF <50%, 0.79 for 50–59%, and 0.82 for ≥60%), the formal test for interaction was not statistically significant (*P* = 0.690), and the confidence intervals for all three subgroups overlapped substantially with the overall estimate (HR 0.80). These findings support the conclusion that the treatment effect is clinically uniform across the LVEF spectrum, with no evidence of meaningful heterogeneity.

These findings support extending the use of SGLT2 inhibitors to HFpEF patients with higher-range LVEF, including those ≥60%, substantially broadening the eligible patient population for this therapy and potentially influencing future guideline refinements. The uniformity of benefit across the LVEF continuum strongly suggests that the cardiorenal protective mechanisms of SGLT2 inhibitors are effective irrespective of the underlying systolic function. This implicates modulation of pathophysiological pathways common to both HFrEF and HFpEF, such as systemic inflammation, sodium-glucose homeostasis, and vascular dysfunction (8).

While our study was not designed to investigate underlying mechanisms, the observed uniform efficacy invites consideration of potential pathophysiological pathways informed by the broader literature. Specific to the HFpEF population, recent analyses detail how these drugs may ameliorate the disorder's core pathophysiology. This is exemplified by the novel ‘adipokine hypothesis’, which reframes HFpEF as a systemic inflammatory disorder originating from adipose tissue—a process that SGLT2 inhibitors may broadly modulate (10). At a more focused mechanistic level, experimental insights suggest that benefits on myocardial metabolism and oxidative stress may be uniquely driven by the inhibition of the SGLT1 receptor, a property of some SGLT2 inhibitors (11). These mechanistic insights, while speculative in the context of our clinical efficacy analysis, provide a plausible framework for understanding the consistent benefits observed across the HFpEF spectrum.

These mechanistic insights, coupled with our consistent efficacy data, provide a compelling foundation for broadening SGLT2 inhibitor use in clinical practice. Specifically, for patients with HFpEF and LVEF ≥60%, the precise risk reduction estimated here (HR 0.82) resolves prior uncertainty and offers a clear basis for treatment initiation. Moreover, the benefit extends robustly to hospitalized or recently hospitalized patients. This supports initiation during or early after the high-risk transition period following a heart failure hospitalization, a strategy aligned with contemporary evidence advocating for therapy optimization in the vulnerable post-discharge phase (12). Importantly, the benefit was evident across the continuum of high-risk status—from patients in a state of recent acute decompensation (as in DELIVER) to those with a marker of chronic high-risk disease (a prior hospitalization within 12 months, as in EMPEROR-Preserved).

Taken together, the consistent efficacy across LVEF strata and high-risk clinical profiles—driven primarily by a robust reduction in heart failure hospitalizations—establishes SGLT2 inhibitors as a foundational therapy for the broad HFpEF population. While the observed neutral effects on cardiovascular and all-cause mortality (HR 0.90 and 0.97, respectively) indicate that the therapeutic profile in HFpEF differs from that in HFrEF, the substantial and consistent reduction in hospitalization burden carries significant clinical implications for both patients and healthcare systems. Consequently, these findings strongly support in-hospital or early post-discharge initiation, a practice further underscored by real-world implementation data (13).

### 4.3 Comparison and innovation in the context of existing evidence

This demonstration of consistent efficacy across the LVEF spectrum challenges the utility of ejection fraction alone for predicting treatment response and prompts a re-evaluation of HFpEF subtyping. While prior meta-analyses have firmly established the overall efficacy of SGLT2 inhibitors in patients with HFpEF (14), they remained underpowered to definitively address heterogeneity across the LVEF spectrum.

Our prespecified, trial-level pooled analysis was designed to directly address this gap regarding LVEF-based heterogeneity. It not only quantifies with precision a consistent treatment effect across all LVEF subgroups (from <50% to ≥60%) but also robustly extends the benefit to clinically important, high-risk populations—specifically, patients recently hospitalized and those with heart failure with improved ejection fraction (HFimpEF), groups in which benefit was initially suggested by the DELIVER trial (4). Consequently, this work synthesizes data directly from the source trials to provide definitive support for a uniform, class-wide therapeutic effect of SGLT2 inhibitors across the broad phenotypic and clinical spectrum of HFpEF.

Traditionally, an LVEF ≥60% has been considered “typical” HFpEF, yet this study demonstrates that the treatment benefit in these patients is comparable to that in those with HFmrEF. This finding supports the evolving trend in current guidelines to manage HFmrEF and HFpEF under a unified therapeutic category (6).

Future research should therefore shift its focus toward actionable phenotypic traits (e.g., obesity, metabolic dysregulation, inflammatory status) rather than relying solely on ejection fraction to predict therapeutic response. This direction is strongly supported by cutting-edge research. For instance, recent large-scale, machine-learning studies have stratified HFpEF into subtypes with distinct clinical profiles, prognoses, and crucially, differential responses to pharmacotherapies (15). Other work has begun to map the dynamic trajectories of these phenotypes between acute decompensation and stable phases (16). These efforts, combined with genetic insights into the fundamental heterogeneity of heart failure (17), collectively support a paradigm shift in managing HFpEF—moving beyond a reliance on left ventricular ejection fraction alone toward a precision medicine framework guided by phenotypic classification.

### 4.4 Ongoing trials and future directions

Several ongoing trials are expected to provide further insights into the role of SGLT2 inhibitors across the HFpEF spectrum and in specific phenotypic subgroups. The DAPA-MODA trial (NCT05893251) is currently investigating the efficacy of dapagliflozin on exercise tolerance and diastolic function in obese patients with HFpEF, a subgroup that may derive particular benefit given the proposed adipokine hypothesis (10). Similarly, the EMPEROR-Preserved 2 trial (NCT05945043) aims to evaluate the long-term effects of empagliflozin on cardiac structure and function in patients with LVEF ≥50%. Additionally, the SGLT2i-HFpEF registry (NCT06012344) is collecting real-world data to assess the effectiveness and safety of SGLT2 inhibitors in routine clinical practice across diverse geographic regions and healthcare settings. The results of these studies, anticipated within the next 2-3 years, will further refine our understanding of which HFpEF phenotypes derive the greatest benefit and may inform more personalized treatment strategies.

Beyond phenotypic refinement, an equally important direction for future research is implementation science. As SGLT2 inhibitors are now indicated for the entire spectrum of HFpEF, studies are needed to identify and overcome barriers to their adoption in real-world practice—including affordability, access to care, health literacy, and long-term medication adherence. Understanding these factors will be essential to ensure that the benefits demonstrated in clinical trials translate into equitable and sustained improvements in patient outcomes across diverse healthcare settings.

### 4.5 Limitations

This study has several limitations. First, this was a trial-level pooled analysis and not an individual patient data analysis, thus precluding adjustment for all potential confounding factors (such as precise NT-proBNP levels). Furthermore, as a pooled analysis of published summary data, we were unable to harmonize differing definitions across trials—for example, “recent hospitalization” was defined as within 30 days in DELIVER but within 12 months in EMPEROR-Preserved. While the consistent treatment effects observed despite these definitional differences support the robustness of our findings, this limitation should be acknowledged. Second, the subgroup analyses rely on data extracted from published trial reports; despite strict adherence to the prespecified protocol, reporting bias may still exist. Third, the analysis of the unique HFimpEF population is based solely on data from the DELIVER trial, requiring validation in future studies. Fourth, the patient populations enrolled in the EMPEROR-Preserved and DELIVER trials were subject to strict inclusion and exclusion criteria. Consequently, our findings may not be fully generalizable to the broader real-world HFpEF population, which often includes older individuals, those with multiple complex comorbidities, frailty, or more advanced renal dysfunction. Future real-world evidence studies and dedicated registries are needed to confirm the effectiveness and safety of SGLT2 inhibitors in these underrepresented populations. Finally, this analysis focuses primarily on hard cardiovascular endpoints; its impact on symptom improvement and quality of life warrants further evaluation.

## 5 Conclusions

In summary, this prespecified pooled analysis definitively demonstrates that SGLT2 inhibitors confer consistent cardiovascular protection in HFpEF regardless of left ventricular ejection fraction (from <50% to ≥60%) or clinical stability status (including recently hospitalized patients and those with HFimpEF). These data resolve prior ambiguities regarding efficacy at higher LVEF and establish a unified “class effect” across the HFpEF spectrum. Consequently, SGLT2 inhibitors should be considered a cornerstone therapy for the broad spectrum of patients with HFpEF, independent of ejection fraction thresholds. This evidence provides a compelling basis for guideline updates and underscores the need for implementation strategies to ensure broad access.

Future research should now pivot from questioning whether to treat based on LVEF, to optimizing how to implement therapy and exploring phenotypic determinants of response within this now-unified therapeutic framework.

## Data Availability

The original contributions presented in the study are included in the article/Supplementary Material, further inquiries can be directed to the corresponding author.
